# Lymphoblastic Leukemia With P2RY8::CRLF2 Rearrangement and High Hyperdiploidy

**DOI:** 10.7759/cureus.90563

**Published:** 2025-08-20

**Authors:** Yui Tsunoda, Hiroko Fukushima, Toru Nanmoku, Takao Deguchi, Hidetoshi Takada

**Affiliations:** 1 Department of Pediatrics, University of Tsukuba Hospital, Tsukuba, JPN; 2 Department of Clinical Laboratory, University of Tsukuba Hospital, Tsukuba, JPN; 3 Division of Cancer Immunodiagnostics, National Center for Child Health and Development, Tokyo, JPN

**Keywords:** acute lymphoblastic leukemia (all), bcp-all, crlf2, high hyperdiploidy, leukemia, p2ry8::crlf2

## Abstract

Improved survival rate in pediatric acute lymphoblastic leukemia (ALL) warrants efforts to optimize treatment intensity in good-prognostic groups such as those with high hyperdiploidy (HHD). Cytokine receptor-like factor 2 (*CRLF2*) alteration is frequently detected in Philadelphia chromosome-like ALL, which has a high relapse ratio and no consensus treatment strategy. *P2RY8::CRLF2*-positive childhood ALL is frequently associated with disease recurrence. To the best of our knowledge, there have been no previous case reports of an ALL patient with *P2RY8::CRLF2* fusion gene, *CRLF2* high expression, and HHD that have included a detailed description of the clinical course. Here we report the case of a 3.3-year-old Sri Lankan boy diagnosed with B-cell precursor (BCP) ALL with *P2RY8::CRLF2*, *CRLF2* hyperexpression, and HHD. The patient’s rapid minimal residual disease (MRD) clearance and HHD karyotype supported classification as standard-risk, per Associazione Italiana di Ematologia Oncologia Pediatrica and the Berlin-Frankfurt-Münster Acute Lymphoblastic Leukemia (AIEOP-BFM ALL2000) criteria. However, the contribution of *CRLF2* overexpression to long-term risk remains uncertain. At 54 months since diagnosis, he remains in good health following his initial complete response.

## Introduction

The prognosis of pediatric acute lymphoblastic leukemia (ALL) has been greatly improved with the development of risk-adapted treatment approaches, and now the five-year event-free survival (EFS) rate is reportedly over 90% for those in low-risk groups [1-3]. To reduce treatment-related adverse events, minimizing the treatment intensity is warranted for good-prognostic groups. However, clinical outcomes are still unsatisfactory in patients with poor prognostic factors, including those with Philadelphia chromosome-positive ALL (Ph-ALL). Patients with leukemic cells with expression patterns similar to Ph-ALL, known as Philadelphia chromosome-like B-cell ALL (Ph-like ALL), have been reported to have poor prognoses [4]. Recent studies report that 22.5% of leukemic cells in Ph-like ALL harbor the *P2RY8::CRLF2* fusion gene [5]. Cytokine receptor-like factor 2 (*CRLF2)* dimerizes with IL-7Rα to form the thymic stromal lymphopoietin (TSLP) receptor, activating JAK-STAT, PI3K, and mTOR pathways. In ALL, *CRLF2* alterations drive constitutive signaling and are linked to chemoresistance, though JAK inhibitors may offer targeted therapeutic options. High *CRLF2* expression typically results from translocations involving *CRLF2* and either *P2RY8* or the immunoglobulin heavy chain (IGH) locus. Alternatively, rare point mutations in *CRLF2* may cause ligand-independent dimerization and activation of downstream signaling [4]. Ph-like ALL accounts for 15-30% of B-cell precursor (BCP)-ALL in older children, adolescents, and adults. Among cases of Ph-like ALL, 22.5% of patients are positive for *P2RY8::CRLF2* [5], and 64% had overexpression of *CRLF2* [6]. Children with BCP-ALL with *P2RY8::CRLF2* fusion experienced high rates of disease recurrence (54-61%); however, sufficient evidence to support changes in treatment strategy, such as upgrading the risk group, has not yet been established [7,8]. On the other hand, high hyperdiploidy (HHD) ALL, defined by a deoxyribonucleic acid (DNA) index of 1.16 or higher, has a good prognosis with a five-year overall survival (OS) rate of over 90% [9,10]. When multiple prognostic factors are present, treatment strategies are usually guided by the factor associated with the worst prognosis. The coexistence of HHD (a favorable marker) and *CRLF2* alterations (a poor-risk feature) creates prognostic ambiguity. Recent evidence suggests that specific trisomies in HHD may ameliorate *CRLF2*-associated relapse risk [11,12]. A previous study with a small sample size showed that patients with Ph-like ALL had similar outcomes to those without Ph-like features when treatment was guided by minimal residual disease (MRD) status [13]. MRD refers to a tumor marker detected by molecular biological methods that identifies residual malignant cells at levels lower than those detectable by conventional microscopy. It is commonly assessed using flow cytometry or polymerase chain reaction (PCR). Furthermore, six cases with t(9;22)(q34;q11)/BCR-ABL1 rearrangement with HHD (0.8% of HHD), all of whom were classified as high risk, were in complete remission, and all but one were alive, suggesting that having HHD may result in a better treatment response than BCR-ABL alone [14]. Currently, there is no established consensus on whether *P2RY8::CRLF2* positivity or high *CRLF2* expression alone could be a poor prognostic factor that warrants a change in treatment strategy. To the best of our knowledge, no prior case report has described an ALL patient with *P2RY8::CRLF2* fusion, high *CRLF2* expression, and HHD with a detailed clinical course. We report a case of childhood BCP-ALL with *P2RY8::CRLF2*, high *CRLF2* expression, and HHD that was successfully treated with a standard-risk protocol, resulting in a favorable outcome.

## Case presentation

The patient was a 3.3-year-old Sri Lankan boy with no previous medical history nor specific family histories of infant deaths or childhood malignancies. After a prolonged cough and arthralgia for one month, he was transferred to our hospital due to hepatosplenomegaly. At the time of his admission, his height was 97.2 cm (+0.6 SD) and his weight was 13.1 kg (-0.6 SD). Physical examination revealed hepatomegaly (4 cm below the costal margin), splenomegaly (extending to the umbilicus), and no testicular enlargement, joint swelling, or purpura. Peripheral blood tests showed leukocytosis with blast cells, anemia, and thrombocytopenia (leukocyte 28.5x10^3^/µL, Hb 5.8 g/dL, platelets 29x10^3^/µL). Blood tests showed mild abnormalities in lactate dehydrogenase (410 IU/L), uric acid (5.2 mg/dL), and coagulation tests (fibrinogen 497 mg/dL) with no electrolyte abnormalities (Tables 1, 2).

**Table 1:** Laboratory findings at admission The bolded values, including leukocytosis with excess blasts, positive chimeric genes, chromosomal abnormalities, and the DNA index, contribute to the stratification of leukemia risk.

Parameter	Value		Reference range
White blood cell count	28.5	×10³ /µL	6.0 – 15.0
Segmented neutrophils	3.5	%	30 – 50
Band neutrophils	0.5	%	0 – 5
Lymphocytes	12	%	40 – 60
Monocytes	0	%	2 – 8
Eosinophils	0	%	1 – 4
Basophils	0	%	0 – 1
Atypical lymphocytes	0	%	0
Plasma cells	0	%	0
Metamyelocytes	0	%	0
Myelocytes	0	%	0
Promyelocytes	0	%	0
Blast cells	84	%	0
Red blood cell count	2.1	×10⁶ /µL	4.0 – 5.0
Hemoglobin concentration	5.8	g/dl	11.0 – 13.5
Hematocrit	18.5	%	33 – 39
Absolute reticulocyte count	29.1	×10³ /µL	20 – 100
Platelet count	29	×10³ /µL	200 – 450
Mean corpuscular volume	88.1	femtoliters	72 – 86
Mean corpuscular hemoglobin	27.6	picograms	24 – 30
Mean corpuscular hemoglobin concentration	31.4	g/dL	32 – 36
Red cell distribution width	17.6	%	11 – 14
Leukemia work-up			
Chimeric-gene screening			
BCR::ABL1	negative		
CBFB::MYH11	negative		
P2RY8::CRLF2	positive		
FLT3-ITD	negative		
FUS::ERG	negative		
KMT2A::AFF1	negative		
KMT2A::MLLT1	negative		
KMT2A::MLLT11	negative		
KMT2A::MLLT3	negative		
KMT2A::MLLT4	negative		
RBM15::MKL1	negative		
RUNX1::RUNX1T1	negative		
TCF3::HLF	negative		
TCF3::PBX1	negative		
Chromosome analysis (G-banding)			
54-55,XY,+X,+4,+6,+9,+10,+14,+18,+21,+21[cp3]			
DNA-index	1.2		

**Table 2:** Blood biochemistry and coagulation

Parameter	Value		Reference range
Total protein	6.6	g/dL	6.0 – 8.0
Albumin	3.7	g/dL	3.8 – 5.0
Aspartate aminotransferase	25	U/L	25 – 45
Alanine aminotransferase	6	U/L	10 – 40
Lactate dehydrogenase	410	U/L	150 – 350
Alkaline phosphatase	100	U/L	300 – 1200
Gamma-glutamyl transferase	10	U/L	8 – 20
Cholinesterase	225	U/L	200-465
Leucine aminopeptidase	40	U/L	20 – 60
Total bilirubin	0.5	mg/dL	0.2 – 1.0
Bile acid	7.1	μmol/L	0 – 10
Sodium	136	mmol/L	136 – 144
Chloride	100	mmol/L	98 – 106
Potassium	4.4	mmol/L	3.5 – 5.0
Blood urea nitrogen	12.8	mg/dL	7 – 18
Creatinine	0.3	mg/dL	0.3 – 0.5
Uric acid	5.2	mg/dL	2.5 – 5.0
Cholesterol	88	mg/dL	130 – 190
Triglyceride	68	mg/dL	30 – 130
Amylase	33	U/L	30 – 110
Lipase	18	U/L	10 – 40
Creatine kinase	15	U/L	30 – 250
Immunoglobulin G	974	mg/dL	600 – 1200
Immunoglobulin A	107	mg/dL	30 – 150
Immunoglobulin M	110	mg/dL	40 – 150
C-reactive protein	2.5	mg/dL	< 0.3
Serum magnesium	2.4	mg/dL	1.8 – 2.4
Calcium	9.4	mg/dL	9.0 – 10.5
Inorganic phosphorus	5.2	mg/dL	4.0 – 6.0
B-type natriuretic peptide	9.4	pg/mL	< 20
Cystatin C	0.61	mg/L	0.6 – 1.0
Coagulation			
Activated partial thromboplastin time (APTT)			
APTT (seconds)	37.3	seconds	25 – 40
APTT (control value)	27.4	seconds	25 – 40
Prothrombin time (PT)			11 – 14
PT (seconds)	12.8	seconds	11 – 14
PT activity	88.1	%	70 – 130
Prothrombin time ratio	1.06		0.9 – 1.1
PT (control value)	11.6	seconds	11 – 14
PT international normalized ratio (INR)	1.07		0.9 – 1.1
Fibrinogen quantitative	497	mg/dL	200 – 400
Antithrombin activity	98.1	%	80 – 120
Fibrin/fibrinogen degradation products	4.6	μg/mL	< 5
D-dimer	1.4	μg/mL	< 1
Protein C activity	64	%	70 – 130
Protein S	49	%	70 – 130

**Figure 1:**
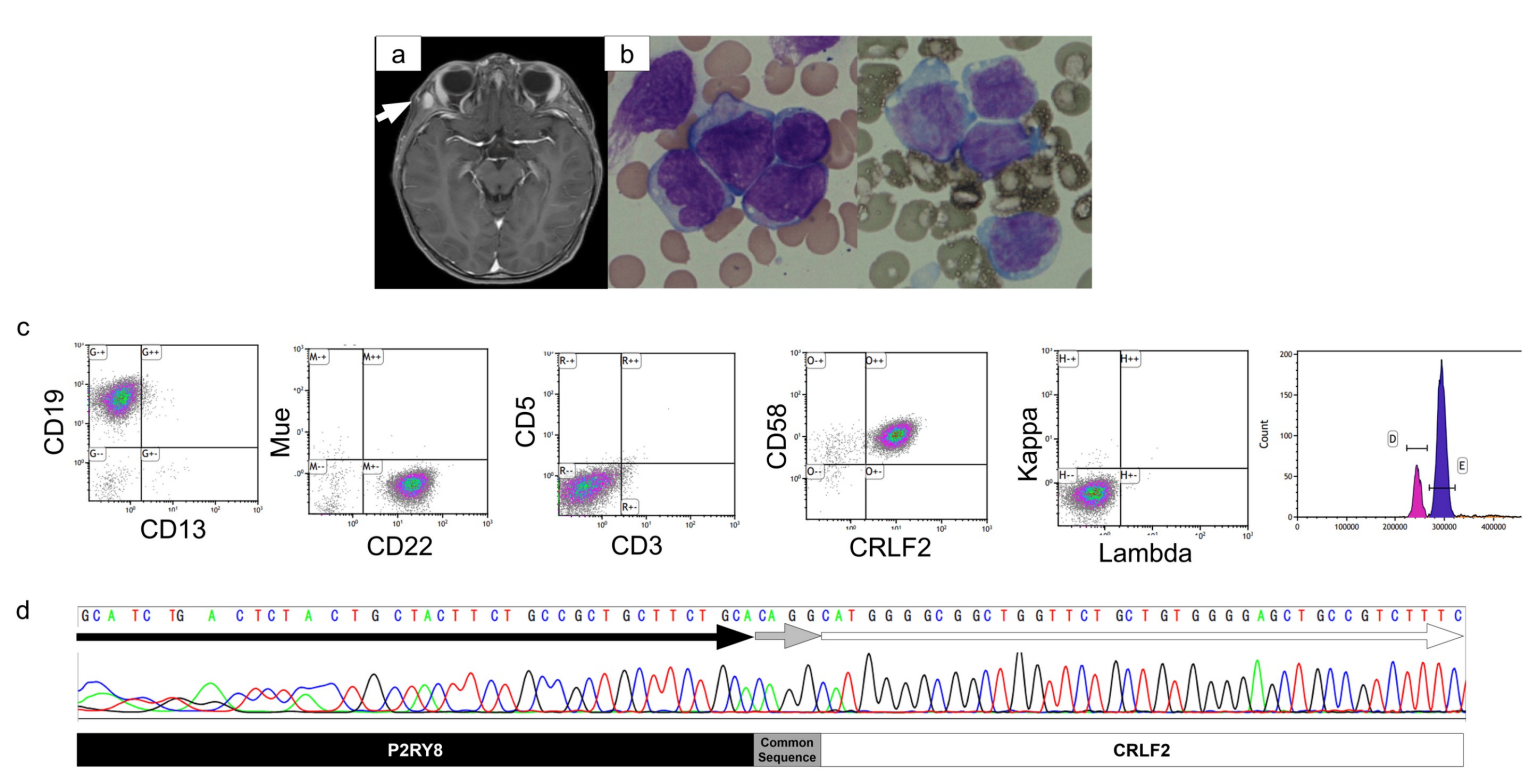
Diagnostic analysis and sequencing data of P2RY8::CRLF2 a: magnetic resonance image of the head and brain showed an 11 mm mass within the right temporalis muscle (arrow); b: bone marrow smear showed blast cells with a large N/C ratio (left) and negative for myeloperoxidase staining (right); c: flow cytometry analysis of peripheral blood showed that the leukemic cell was a B-cell precursor, *CRLF2* positive, and high-hyper diploidy; d: sequencing data of *P2YR8::CRLF2* chimeric gene.

Analysis of cerebrospinal fluid and contrast-enhanced magnetic resonance image (MRI) showed no infiltration of leukemic cells into the central nervous system. An extramedullary nodule of 11 mm in size was detected in the right temporal muscle (Figure 1a).

Bone marrow aspiration showed an increase in lymphoblastoid cells that were negative for myeloperoxidase staining (Figure 1b). Flow cytometry showed that leukemic cells were positive for CD19, 22, and *CRLF2*, and negative for CD3, CD5, Igκ, and Igλ. The patient was diagnosed with BCP-ALL with high *CRLF2* expression. DNA index analysis revealed HHD of 1.20 concurrent with *CRLF2* (Figure 1c). Chimeric gene screening revealed a *P2RY8*(exon6)-*CRLF2*(exon3) fusion gene (Figure 1d). Chromosomal analysis of G-band staining showed an increase in the number of chromosomes as follows: 55, XY, +X, +4, +6, +9, +10, +14, +18, +21, +22 [4]/46, XY [6].

The patient underwent treatment according to the Associazione Italiana di Ematologia Oncologia Pediatrica and the Berlin-Frankfurt-Münster Acute Lymphoblastic Leukemia (AIEOP-BFM ALL2000) protocol [1]. The number of blasts in peripheral blood was markedly decreased to 0.28x10^9^/L on day eight after prephase treatment with prednisolone (PSL), and the patient was determined to be a PSL good responder.

**Figure 2:**
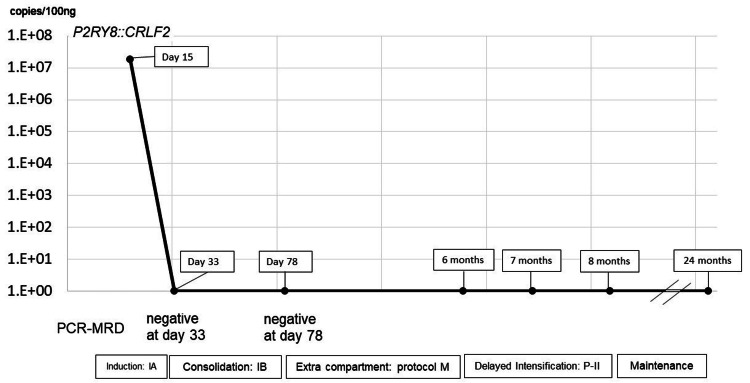
Status of the minimal residual disease during therapy The chimeric gene copy number and polymerase chain reaction-based minimal residual disease status are illustrated along the course of treatment.

After the induction phase IA therapy and induction consolidation phase IB, bone marrow aspiration revealed complete bone marrow remission, and, on PCR, there was no detectable MRD or *P2RY8::CRLF2* chimeric gene, and no extra medullary lesions in the right temporal muscle (Figure 2).

The patient was treated according to the standard-risk arm of the AIEOP-BFM ALL2000 protocol, consisting of prephase with prednisone 60 mg/m² on days one to seven and intrathecal methotrexate on day one; induction IA phase with vincristine 1.5 mg/m² on days eight, 15, 22, and 29, prednisone 60 mg/m² daily from days eight to 28, daunorubicin 30 mg/m² on days eight, 15, 22, and 29, L-asparaginase 5,000 IU/m² on days 12, 15, 18, 21, 24, 27, 30, and 33, and intrathecal methotrexate on days 12 and 33; consolidation IB phase with cyclophosphamide 1,000 mg/m² on days 36 and 64, 6-mercaptopurine 60 mg/m² daily from days 36 to 63, cytarabine 75 mg/m² on days 38 to 41, 45 to 48, 52 to 55, and 59 to 62, and intrathecal methotrexate on days one, 12, and 33; protocol M with 6-mercaptopurine 25 mg/m² daily from days 1 to 56, high-dose methotrexate 5 g/m² on days eight, 22, 36, and 50, and intrathecal methotrexate on the same days; reinduction phase (protocol II) with dexamethasone 10 mg/m² on days one to 21, vincristine 1.5 mg/m² on days eight, 15, 22, and 29, doxorubicin 30 mg/m² on days eight, 15, 22, and 29, L-asparaginase 10,000 IU/m² on days eight, 11, 15, and 18, cyclophosphamide 1,000 mg/m² on day 36, 6-mercaptopurine 60 mg/m² daily from days 36 to 49, cytarabine 75 mg/m² on days 38 to 41 and 45 to 48, and intrathecal methotrexate on days 38 and 45; followed by a maintenance phase with continued administration of 6-mercaptopurine and methotrexate. After 24 months of treatment since initial diagnosis, including 16 months of maintenance therapy, the patient remains in continuous molecular remission (MRD-negative at four-month intervals) for 54 months from diagnosis. No treatment-related toxicities or secondary malignancies were observed.

## Discussion

Here, we reported a Sri Lankan boy diagnosed as BCP-ALL with *P2RY8::CRLF2* rearrangement, high *CRLF2* expression, and HHD. Although *P2RY8::CRLF2* rearrangement has been reported to be more common in Native American or American Hispanic populations [4], there have been no notable reports of *P2RY8::CRLF2 *cases in Sri Lankan population. The lack of previously reported cases in Sri Lankan individuals may reflect ethnic differences, disparities in access to medical diagnostics, or challenges related to case reporting and publication. Continued monitoring of future studies will be essential to clarify this issue.

Although unfavorable prognostic factors are generally given precedence when both favorable and unfavorable markers are present, the simultaneous detection of the *P2RY8::CRLF2* fusion gene, whose prognostic significance remains insufficiently validated-and the favorable HHD complicated clinical decision-making in this case. Five-year OS and EFS vary among ALL cases with HHD, *P2RY8::CRLF2*, and high *CRLF2* expression, even when diagnosed at similar ages [4,15]. Our case was diagnosed at 3.3 years old, which is in line with previous reported ALL cases that involve either *P2RY8::CRLF2*, high *CRLF2* expression, or HHD. In cases of BCP-ALL, HHD reportedly occurs in 25-30% [9,15], *P2RY8::CRLF2* in 6%, high *CRLF2* expression in 4.7%, and concurrent *P2RY8::CRLF2* and high *CRLF2* expression in 2-3% [8]. The five-year OS rates of patients with *P2RY8::CRLF2* rearrangement, high *CRLF2* expression, and HHD are 80.9-93.7%, 85.9%, and 89-96%, respectively [8-10]. The five-year EFS of patients with *P2RY8::CRLF2* rearrangement, high *CRLF2* expression, and HHD are 57.2%, 62.9%, and 83-93.7%, respectively [1,8,10]. The five-year EFS for patients with both *P2RY8::CRLF2* and high *CRLF2* expression is 37.5% [8]. Thus, while patients with *CRLF2* alterations have a high relapse ratio, treatment for the relapse is often successful. To our knowledge, currently, there are no data on the frequency or survival outcomes for patients with concurrent *P2RY8::CRLF2* rearrangement, high *CRLF2* expression, and HHD. Negative MRD status after induction therapy, and the percentage of patients who underwent standard risk protocol was 44.8% for HHD, 27.3% for *P2RY8::CRLF2*, and 13.6% for high *CRLF2* expression, respectively [1,3]. MRD in our patient showed a rapid decrease and was negative at the completion of induction therapy, which supports our strategy to continue to treat as a standard risk. Although no comprehensive reports have been published regarding the discrepancy between chimeric gene detection and PCR-MRD assessment, it is presumed that both methods may yield false-negative results or discordance with the actual presence of residual leukemic cells due to factors such as sample handling and PCR efficiency. Chimeric gene detection, which uses ribonucleic acid (RNA) as a template, may reflect the transcriptionally active state of tumor cells, whereas PCR-MRD, which utilizes more stable DNA, may more accurately reflect the actual number of residual leukemic cells. PCR-MRD is a highly versatile method that can be applied to most of the BCP-ALL cases, and its utility in stratifying treatment response has been well established in previous studies [1]. In contrast, chimeric genes are detected in only approximately one-fourth of pediatric BCP-ALL cases [16]. Although the precise functional role of the *P2RY8::CRLF2* fusion gene to be leukemia remains fully understood, it is presumed, unlike ETV6::RUNX1, that the presence of this genetic alteration alone is sufficient to reflect leukemic transformation rather than representing a preleukemic or non-transforming event [17]. Accordingly, detection of the *P2RY8::CRLF2* fusion is likely indicative of residual leukemic cells. However, there is currently no established evidence regarding the threshold levels of this fusion transcript that may be considered acceptable during each point of therapy. In our case, if there had been a discrepancy between *P2RY8::CRLF2* fusion transcript levels and PCR-MRD results, we would have prioritized treatment decisions based on PCR-MRD, given its higher level of clinical validation [1]. Nonetheless, considering the potential for false-negative results in both assays, if PCR-MRD were negative but the *P2RY8::CRLF2 *transcript levels failed to decline, we would have remained vigilant for possible treatment resistance. In such a scenario, we may have considered treatment intensification depending on the overall clinical context.

Relapses in patients with *P2RY8::CRLF2* or high *CRLF2* expression are reported to occur after the end of the two-year treatment [8]. Our patient was alive without relapse 54 months after diagnosis. 

In two previous studies, HHD was detected in 16% (six of 38 patients), 23% (five of 22 patients) [8,18] with high *CRLF2* expression, and zero of 22 patients were positive for *P2RY8::CRLF2* [8]. In one of these studies, of the six patients with concurrent HHD and high *CRLF2* expression, only one experienced relapse, which corresponded to the standard risk group [18]. We have not found any previously reported cases of ALL with concurrent high *CRLF2* expression, *P2RY8::CRLF2* chimeric genes, and HHD. Although there is no established treatment strategy for ALL with concurrent *CRLF2* deregulation and HHD, it has been reported that when patients with BCR-ABL1-like ALL were treated according to their MRD status, the prognosis was not different between patients with and without BCR-ABL1-like ALL [13]. Our patient underwent standard risk protocol according to his MRD status. He is alive without relapse 54 months after diagnosis with ongoing observation and follow-up. The clinical impact of overlapping *P2RY8*::*CRLF2* rearrangement and hyperdiploidy in BCP-ALL remains to be fully understood and warrants further multi-institutional documentation.

Our patient had an extramedullary lesion in the temporalis muscle and became undetectable at the end of induction therapy. The patients with extramedullary lesions are recognized to be higher risk than those without [19]. However, other than the central nervous system or testicular lesion, its effect on the clinical course is relatively uncertain due to its rarity. 

This report has several limitations. First, it describes only a single case, which is insufficient to draw generalized conclusions. In addition, data on *CRLF2*-altered ALL in South Asian populations remain limited, and most available studies are based on Western cohorts. Therefore, it cannot be assumed that the findings observed in predominantly Western populations apply equally to individuals of different ethnic backgrounds.

## Conclusions

A 3.3-year-old Sri Lankan boy diagnosed as having BCP-ALL with concurrent *P2RY8::CRLF2* rearrangement, *CRLF2* high expression, and HHD. Although he had contraindicated prognostic factors, he underwent the standard risk treatment according to the MRD-based strategy. He is in a good clinical course at 54 months after diagnosis.
